# Chemical LTP induces confinement of BDNF mRNA under dendritic spines and BDNF protein accumulation inside the spines

**DOI:** 10.3389/fnmol.2024.1348445

**Published:** 2024-02-21

**Authors:** Giorgia Bimbi, Enrico Tongiorgi

**Affiliations:** Department of Life Sciences, University of Trieste, Trieste, Italy

**Keywords:** brain-derived neurotrophic factor, mRNA trafficking, local protein synthesis, synaptic plasticity, dendritic spines, live imaging

## Abstract

The neurotrophin brain-derived neurotrophic factor (BDNF) plays a key role in neuronal development and synaptic plasticity. The discovery that BDNF mRNA can be transported in neuronal dendrites in an activity-dependent manner has suggested that its local translation may support synapse maturation and plasticity. However, a clear demonstration that BDNF mRNA is locally transported and translated at activated synapses in response to long-term potentiation (LTP) is still lacking. Here, we study the dynamics of BDNF mRNA dendritic trafficking following the induction of chemical LTP (cLTP). Dendritic transport of BDNF transcripts was analyzed using the MS2 system for mRNA visualization, and chimeric BDNF-GFP constructs were used to monitor protein synthesis in living neurons. We found that within 15 min from cLTP induction, most BDNF mRNA granules become stationary and transiently accumulate in the dendritic shaft at the base of the dendritic spines, while at 30 min they accumulate inside the spine, similar to the control CamkIIα mRNA which also increased inside the spines at 60 min post-cLTP. At 60 min but not at 15 min from cLTP induction, we observed an increase in BDNF protein levels within the spines. Taken together, these findings suggest that BDNF mRNA trafficking is arrested in the early phase of cLTP, providing a local source of mRNA for BDNF translation at the base of the spine followed by translocation of both the BDNF mRNA and protein within the spine head in the late phase of LTP.

## 1 Introduction

Morphological changes in dendritic spines have consistently been observed during the process leading to the establishment of long-term synaptic changes and memory engrams (Zagrebelsky et al., 2020). The mechanisms underlying these structural changes have been largely elucidated by studies over the last two decades. In particular, there is now evidence that the enlargement of the spine head that accompanies synaptic long-term potentiation (LTP), a cellular correlate of memory formation, is entirely dependent on protein synthesis and the availability of neurotrophic factors such as the neurotrophin brain-derived neurotrophic factor (BDNF) [reviewed in Panja and Bramham (2014), Leal et al. (2017), and Zagrebelsky et al. (2020)]. In particular, an early study showed that in order for LTP to be induced, BDNF must be released locally at synapses (Tanaka et al., 2008). A more recent study indicated that when BDNF is released from postsynaptic terminals, LTP can be induced by activating an autocrine loop through TrkB receptors located on the very same postsynaptic spine compartment from which BDNF is released (Harward et al., 2016). Nevertheless, despite all these studies, the question of whether the postsynaptically released BDNF is synthesized locally in this compartment or has a different origin remains unanswered (Leal et al., 2017; Song et al., 2017).

Studies in rodent hippocampal cultures have shown that BDNF is released preferentially from dendritic compartments in an activity-dependent manner upon Ca^2+^ entry through postsynaptic NMDA receptors (Kuczewski et al., 2009; Matsuda et al., 2009). It is therefore logical to assume that in response to LTP induction, BDNF is synthesized in dendritic spines or the dendritic shaft close to the activated synapses. However, there is also evidence that BDNF can be endocytosed at the postsynaptic level and further released after LTP induction (Santi et al., 2006; Wong et al., 2015). Surprisingly, studies using a genetically modified mouse in which endogenous BDNF was HA-tagged reported the presence of this chimeric BDNF only in the presynaptic compartment (Matsumoto et al., 2008; Dieni et al., 2012), reviewed in Song et al. (2017). On the other hand, in support of the model posing that BDNF also has a postsynaptic localisation, several research groups have gathered a large body of evidence at functional, immunofluorescence and electron microscopy levels demonstrating BDNF localisation within post-presynaptic terminals (Edelmann et al., 2015; Harward et al., 2016; Song et al., 2017; Brigadski and Leßmann, 2020).

Additional support for the view that BDNF localises to postsynaptic terminals has come from our own and other group studies showing that BDNF mRNA is present in dendrites, but not in axons, and that its transport to the distal dendritic compartment is increased *in vivo,* in response to seizures, antidepressants, and physical activity (Tongiorgi et al., 2004; Baj et al., 2012), and *in vitro*, in response to electrical activity and treatment with NT3 or BDNF itself (Tongiorgi and Baj, 2008; Oe and Yoneda, 2010; Mallei et al., 2015; Vicario et al., 2015; Lekk et al., 2023). Furthermore, chimeric BDNF-GFP constructs expressed in cultured neurons exhibit local translation in dendrites in response to electrical activity (Baj et al., 2011; Vaghi et al., 2014; Lekk et al., 2023), and in experimental animals, endogenous BDNF protein and mRNA have an overlapping distribution in the hippocampal laminae containing the dendrites, both in resting conditions and following various types of stimuli (Tongiorgi et al., 2004; Baj et al., 2012). Despite these findings, it remains unclear whether BDNF is translated locally in dendrites or within the spine.

Several mRNAs coding for various classes of proteins are known to be targeted to dendrites in mature neurons (Holt et al., 2019). Among them, there are proteins of synaptic elements, translation factors, cytoskeleton, RNA-binding proteins, and components of signaling pathways, including neurotrophic factors such as BDNF (Holt et al., 2019; Glock et al., 2021). Recent studies have demonstrated that abundant dendritic mRNAs, including the one encoding calmodulin kinase II (CaMKII) or Rgs4, can move in both forward and backward directions. However, following stimulation of the cultures, they tend to become stationary in dendrites and may eventually be locally translated in the vicinity of activated synapses (Bauer et al., 2019; Donlin-Asp et al., 2021). An insightful review article by Rangaraju et al. (2017) put forward the question of how strictly compartmentalized the local production of a protein arising from the translation of a dendritic mRNA is. In other terms, is there a widespread translation of an mRNA, spanning a large segment of a dendrite, or is it a phenomenon restricted to the immediate neighborhood of a spine?

In this study, we investigate these unresolved questions by tracking the trafficking of BDNF mRNA within dendrites and its local translation following the induction of chemical LTP (cLTP) in living mouse hippocampal neurons. This was done by using chimeric constructs that were ectopically expressed in primary hippocampal cultures.

## 2 Materials and methods

### 2.1 Primary hippocampal neuronal cultures

The animal study was approved by Organismo Preposto al Benessere degli Animali (OPBA) of the University of Trieste and the Italian Ministry of Health. The study was conducted in accordance with the local legislation and institutional requirements. Dissociated hippocampal neurons were prepared from P0-P1 C57BL6/J mice pups. Hippocampi were dissected out in cold HBSS medium (Minimal Essential Medium, 0.2% MOPS, 20 mM glucose, 1 mM sodium pyruvate) and then incubated in 300 μL of 0.25% trypsin in HBSS at 37°C for 8 min. After 5 min of centrifugation at 8000 rpm, cells were mechanically dissociated in DMEM (DMEM 1x +5% of FBS) by pipetting up and down for 10 times with a Pasteur pipette. Cells were counted with the dye exclusion method using Trypan Blue (Sigma) in the Burker chamber (Eppendorf), and 400,000–500,000 cells were obtained from each mouse. Cells were plated either on 12 mm cover slides coated with 0.1 mg/mL poly-L-ornithine (Sigma) or on μ-Slide 8 Well Glass Bottom (#1.5 polymer coating cat. No. 80806 from IBIDI) at a density of 130,000 cells/well. To reach this density, having different coverslips per experiment, a pool of hippocampi from different animals was used for each preparation. After 1 h from plating, DMEM was replaced with Neurobasal medium supplemented with 2% B-27 (Invitrogen), 1 mM Ala-Gn (Glutamax, G1845, Sigma), 0.45% glucose, and 1% penicillin–streptomycin. A final concentration of 1.25 uM Ara-C was added at days *in vitro* 4 (DIV4) to inhibit glia proliferation. Neurons were cultured under a humidified 5% CO_2_ atmosphere at 37°C for 14 days. At DIV4, half medium was replaced by adding Ara-C. Neurons were transfected in 8 μ-Slide 8 Well Glass Bottom chamber slides (IBIDI) with 1.2 μL of DNA and 1.2 μL Lipofectamine 2000 per well and 50 μL of MEM. To study the BDNF protein, transfections of EX6-BDNFcds-gfp-3 L or Ex1-BDNFcds-gfp-3 L (CMV promoter, pEGFP-N1 backbone, previously described in Baj et al., 2011) were carried out at DIV9, and cultures were visualized at DIV13-14. To study BDNF mRNA trafficking, neurons were transfected between DIV11-13 with plasmids derived from the previous BDNF constructs made in the pEGFP-N1 backbone (Baj et al., 2011), in which we replaced the EGFP with the 12-loop insert, and dynamic of mRNA granules was analyzed at 10–24 h post-transfection. Specifically, transfections were made with the following plasmid pairs: EX6-BDNFcds-12xMS2-3 L (CMV promoter, pEGFP-N1 backbone, GenScript) and MS2-NLS-mcherry (CMV promoter, pcDNA 3.1 backbone); Ex1-BDNFcfds-12xMS2-3 L (CMV promoter, pEGFP-N1 backbone, GenScript) and MS2-NLS-mcherry; CaMKIIa-12xMS2-3’UTR (vector RSV-lacZ-MS2bs-CaMKIIα 3′UTR, containing the mouse coding sequence) and the 3’UTR of CaMKIIα, a kind gift of Kenneth Kosik (Rook et al., 2000) together with MS2-NLS-mcherry.

### 2.2 cLTP protocols and solutions

Long-term potentiation was chemically induced by incubation for 30, 60, and 90 min with 10 μM Forskolin (Sigma 344,270) in a solution containing 92.4 mM NaCl, 2.3 mM KCl, 1.3 mM CaCl_2,_ 0.35 mM Na_2_HP0_4,_ 4.2 mM NaHCO_3,_ 0.45 mM K_2_HPO_4,_ 7 mM HEPES, and 5.5 mM D-glucose. The control solution contained 92.4 mM NaCl, 2.3 mM KCl, 0.8 mM MgSO_4,_ 1.3 mM CaCl_2,_ 0.35 mM Na_2_HP0_4,_ 4.2 mM NaHCO_3,_ 0.45 mM K_2_HPO_4,_ 7 mM HEPES, and 5.5 mM D-glucose. The pH was 7.4. Solutions were pre-warmed before the experiments. After cLTP induction, neurons were fixed for 15 min at room temperature (RT) with 4% paraformaldehyde, pH 7.2. Cells were then permeabilised with 0.01% Triton X-100 in PBS for 15 min. Non-specific binding sites were blocked with 2% BSA in PBS 0.01% Triton X-100 for 30 min. Then, neurons were incubated in the same solution with rabbit anti-c-fos (Sigma # F7799, dil. 1:1000) for 120 min at RT or overnight at 4°C. After washes in PBS, neurons were incubated for 90 min with anti-rabbit Alexa 488 secondary antibodies (made in donkey, dil. 1:500. Thermo Fisher Scientific # A11034) diluted in blocking solution. After washes with PBS, the coverslips were incubated for 5 min with Hoechst 1:1000 to mark the nuclei. The coverslips were then washed in water and mounted using Moviol. Once the immunostaining for c-fos was concluded, the images of neuronal cultures were acquired with a Nikon Eclipse Ti-E-epifluorescence microscope using a 20x objective. Through a specific MACRO created using the NIS software element, between 6 and 10 fields of 1636*1088 pixels were acquired for each coverslip in two channels (DAPI and 488 for the c-fos signal). The exposure time was 200 ms. The images were then analyzed using a MACRO created in Fiji. The MACRO consists of considering DAPI-positive cells that are inside a specific range (between 5 and 10 μm), creating a mask on them and then exporting the mask on the c-fos channel to measure the intensity of the c-fos. The different steps to build the MACRO are presented in Supplementary Figure S1.

### 2.3 KCl treatment

A positive control for the neuronal stimulation was the KCl. Different KCl solutions were used. As a positive control, 50 mM KCl for 180 min was used (Baj et al., 2016). Then, neuronal cultures were incubated for 90 min with solutions containing KCl at 10 mM, 20 mM, and 50 mM and other components, as indicated in Supplementary Table S1.

### 2.4 Live imaging protocol for the BDNF mRNA granules

Neurons plated in IBIDI chamber slides (IBIDI GmbH, Germany) were transfected as described at point 2.1 with EX6-BDNFcds-12xMS2-3 L and MS2-NLS-mcherry or Ex1-BDNFcfds-12xMS2-3 L and MS2-NLS-mcherry, and time-lapse video images were taken 17–18 h after transfection. To acquire movies, a Nikon Eclipse Ti-E-epifluorescence microscope using a 40Xobjective (1.0 NA oil PlanApo DICH) equipped with a DS-Qi2 Camera (NIKON) was used. Movies of 10 min at 1 frame/s for the control condition and 15 min at 1 frame/s for the cLTP condition were acquired. During the 10 min of control recordings, neurons were in the control medium (as described at point 2.2). Then, a wash with the medium without MgSO4 was done. For stimulating neurons, 10 μM of forskolin was added to the medium without MgSO4, and neurons were imaged for a further 15 min.

### 2.5 BDNF protein transfection and visualization in live

Neurons coated in IBIDI chamber slides were transfected with ex1-BDNFcds-GFP-3’UTR long or ex6-BDNFcds-GFP-3’UTR long or BDNFcds-GFP. To visualize the dendrites and the dendritic spines, neurons were co-transfected with an mCherry filler. Video time-lapse images were taken at 17–18 h from transfection. Nikon Eclipse Ti-E-epifluorescence using a 40X objective (1.0 NA oil PlanApo DICH) equipped with a DS-Qi2 Camera (NIKON) was used to acquire the movies. Recordings of 10 min at 1 frame/min for the control condition and 60 min at 1 frame/min for the activated condition were acquired. During the 10 min of control condition, neurons were in the control medium (as described at point 2.2.). Then, a wash with the medium without MgSO4 was done. For stimulating neurons, 10 μM of forskolin was added to the medium without MgSO4, and neurons were imaged for a further 60 min.

### 2.6 Video time-lapse analysis for the BDNF mRNA and protein

After the acquisition, movies were opened on ImageJ (NIH), and kymographs were extracted using the plugin Multi Kymograph. Once the Kymographs were extracted, the analysis consisted of selecting a particular movement of the granules by drawing a segmented line over the corresponding track of the kymograph. Then, due to a custom ImageJ MACRO, the position and the time coordinates (x and t, respectively) from the segmented line were extracted. Thus, all the necessary information about the dynamics of the granules, such as the average and instantaneous velocities and the overall behavior of the particle (anterograde, retrograde or confined), was extracted. The net movement of a granule was considered. Confined movements were defined for granules that moved less than 0.5 μm. For the video of the BDNF protein, only neurons transfected with BDNFcds-GFP were then analyzed because with other constructs neurons were not well visible. The mean fluorescence of BDNFcds-GFP protein spots was measured every 10 min during the 60 min of Forskolin treatment.

### 2.7 Evaluating the distance between a granule and a synapse

After 17–18 h from transfection with BDNF mRNA, neurons were stimulated with 10 μM Forskolin for 15, 30, and 60 min. Then, neurons were fixed and labeled with anti-Synapsin I antibody (Rabbit, dil. 1:1000, Millipore cat. no. AB1243) and revealed by anti-rabbit secondary antibodies (Alexa 488 made in goat, dil. 1:500; Thermo Fisher Scientific, cat. No. A11034). The images were acquired under the Elyra 7 microscope at 63X objective using a filter combination: BP 420–480 and BF 405/488/561/642. SIM images were reconstructed through the Zen-black software. A SIM reconstruction followed by a Z-stack projection was performed using Zen-black software. A threshold for the channel of Synapsin I was applied, and a mask was created. As the green channel was already used for Synapsin I, the same red channel was used to visualize both the BDNF mRNA granules in red and the mCherry filler, which was post-processed in blue, resulting in a magenta color when the red granules overlapped with the blue filler. This strategy was feasible because the fluorescence intensity of the filler was much lower than that of the granules, so the two signals were clearly distinguishable, and two thresholds were considered: one to identify the granules and a second to delineate the structure of the neuron with the associated spines. A MACRO for speeding up the analysis was made. Once the thresholded and masked images were created, stretches of neurons between 30 and 50 μm with visible spines were considered. Granules were counted between 3 μm (away from the soma) and -3 μm (closer to the soma) from a longitudinal axis that divides into two parts the spine. So, all granules within this interval of a total of 6 μm were considered. Granules inside the spines were also counted. Stretches of 30–50 μm were considered of apical distal dendrites.

### 2.8 Statistical analysis

Data analysis was performed blind for all experiments. Statistical values are represented as mea*n* ± S.E.M when data are parametric. When data are not parametric, media*n* ± 95% confidence was chosen. The number of experiments and cells analyzed for each situation is indicated in each figure. Statistical significance was calculated using GraphPad Prism version 8 (GraphPad Software, La Jolla, United States). The normality distribution test was done by Shapiro–Wilk’s test. If samples proved to have a normal distribution, then Student’s *t*-test (for two groups) or ANOVA with Tukey’s post-test (for three or more groups) was used. When samples did not have a parametrical distribution, then for experimental conditions with two data sets, the Mann–Whitney test was used or the Kruskal–Wallis for three or more groups. χ^2^ statistical analysis was also performed to identify independence between categories. Cumulative distribution of data was also used. The outliers were removed using the formula with the interquartile range rule. This was required to calculate the first quartile *Q*_1_ and the third quartile *Q*_3_ and the difference between them. The difference was then multiplied by 1.5. It was necessary to add 1.5x (*Q*_3−_ *Q*_1_) to the third quartile and to subtract 1.5x (*Q*_3−_ *Q*_1_) any number greater or smaller than these values was a suspected outlier. Significance was set as *p < 0.05, **p < 0.01, and ***p < 0,001. The value of p is reported in each figure legend in the Result section as the statistics used for each experiment.

## 3 Results

### 3.1 Time course for the visualization of exogenous mRNA after transfection

**Figure 1:**
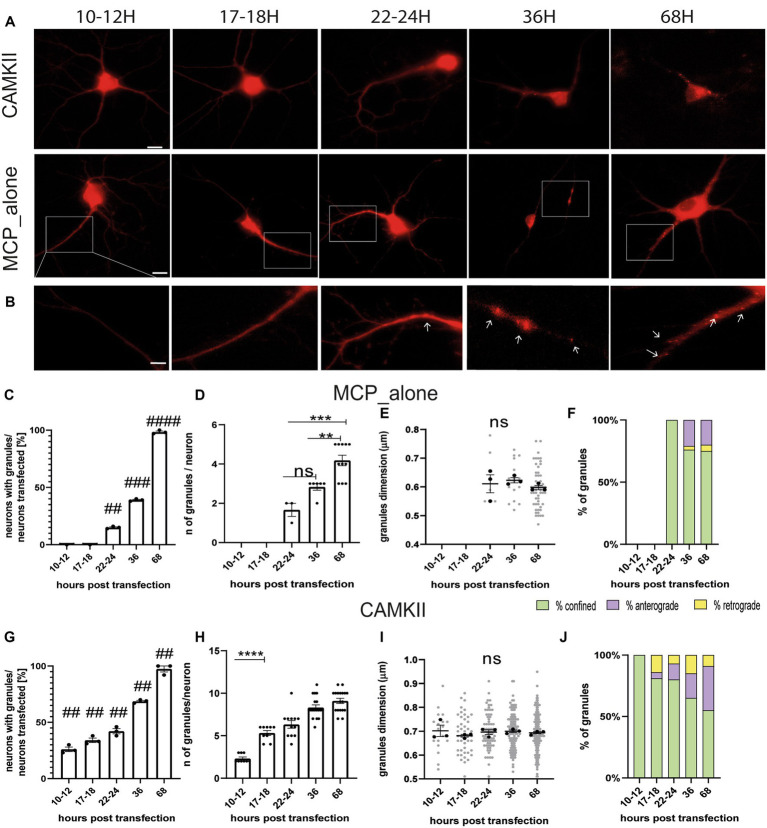
Settings for live-imaging analysis. **(A)** Representative images of MCP_alone and CamkIIα in hippocampal neurons at different times post-transfection. **(B)** High-magnification images of neurons transfected with MCP_alone. Arrows indicate granules. **(C)** Number of neurons with granules after different times post-transfection (*t*-test). **(D)** Number of granules present in the neurons transfected after different times. Note that before 22–24 h, granules were not detected (ANOVA followed by Tukey’s post-hoc test). **(E)** Dimension of the MCP_granules (ANOVA followed by Tukey’s post-hoc test). **(F)** Movement dynamics of granules (*χ*^2^ test). **(G)** Number of neurons with granules after different times post-transfection (*t*-test). **(H)** Number of granules present in the neurons at the different time points from transfection. **(I)** Dimension of CamkIIα granules (ANOVA followed by Tukey’s post-hoc test). **(J)** CamkIIα movement dynamics after different hours post-transfection (*χ*^2^ test; *p* < 0.001 at 10–12 and 17–18 h post-transfection). For each graph, *n* = 15–45 neurons from 3 independent experiments were analyzed.

The MS2 loop system is a well-known method for imaging mRNA trafficking in living neurons (Dahm et al., 2008). The technique involves inserting a repeat of identical stem-loop structures, derived from bacteriophage RNA, into a gene of interest. This is accompanied by the ectopic expression of fluorescently tagged MS2 coat-binding protein (MCP), which binds to the MS2 loops. Previous studies have reported that overexpression of MCP can potentially generate artefactual protein aggregates (Dahm et al., 2008; Yoon and Gorospe, 2016; Bauer et al., 2019). Therefore, in the first set of experiments, we conducted a time course analysis to determine the optimal time point for imaging neurons and avoid possible artefacts. Neurons were co-transfected at DIV 11–12 with two plasmids. The first plasmid encoded the positive control gene CamkIIα, which bore eight MS2 loops between the coding region and its 3’untranslated region (CamKII-8 L-3’UTR). The second plasmid encoded the MS2 coat protein (MCP) fused with the fluorescent reporter gene mCherry (MCP-mCherry). As a negative control, neurons were transfected with only the plasmid encoding MCP-mCherry. Neurons were imaged at different time points from transfection, i.e., 10–12, 17–18, 22–24, 36, or 68 h (h; Figures 1A,B). In all conditions, 10-min-long videos were recorded for each neuron, and the movements of fluorescent spots were analyzed. In control experiments, MCP-mCherry protein spots were detected starting from 22 to 24 h and were not visible at earlier times (MCP_alone, Figure 1C). The number of neurons with MCP-mCherry protein aggregates increased significantly over time (Figure 1C). Specifically, 15% of neurons showed protein spots at 22–24 h (## *p* = 0.0015 vs. 10–12 h), 40% of neurons after 36 h (### *p* = 0.0002 vs. 10–12 h), and 100% after 68 h from transfection (#### *p* ≤ 0.0001 vs. 10–12 h, *n* = 3 independent cultures). The number of MCP-mCherry protein aggregates detected in each neuron (Figure 1D) also increased over time, from an average of 1.68 protein spots/neuron (22–24 h) to 2.83 protein spots/neuron (36 h) and 4.18 protein spots/neuron (68 h). The ANOVA test followed by Tukey’s multiple comparisons indicated that this increase was significant only at later stages (22–24 h vs. 36 h *p* = 0.0910, ns, 22–24 h vs. 68 h *p* = 0.0002 ***; 36 h vs. 68 h *p* = 0.0056 **). Of note, the diameter of the spots, taken as a measure of the size of the MCP-mCherry protein aggregates, remained unchanged over time (Figure 1E; 22–24 h vs. 36 h *p* = 0.89; 22–24 h vs. 68 h *p* = 0.88; 36 h vs. 68 h *p* = 0.64).

Live imaging analysis was conducted to evaluate the dynamics of MCP-mCherry protein aggregates using ImageJ (Multi Kymograph plugin). Depending on the observed movement, each protein spot was classified as either anterograde (moving prevalently away from the cell body), retrograde (moving toward the cell body), or confined protein spots (with movements resulting in a total displacement of less than 0.5 μm). Live-cell videos were acquired at 1 frame/s using a 40X oil objective. Dynamic analysis showed that MCP-mCherry protein spots displayed a different percentage of confined, anterograde, or retrograde behavior at different time points (*χ*^2^ test *p* < 0.0001) (Figure 1F). Specifically, at 22–24 h, 100% of MCP-mCherry protein aggregates were confined; at 36 h, protein spots were 76% confined, 21% anterograde, and 3% retrograde, and at 68 h protein spots were 75% confined, 20% anterograde, and 5% retrograde. Each plot displays the results of *n* = 15–45 neurons from 3 independent cultures (5–15 neurons, each). Taken together, these findings suggest that imaging of neurons should be conducted no earlier than 22–24 h after transfection to avoid the artefact caused by the aggregation of the MCP protein alone. Therefore, the preferable time windows to image neurons are 10–12 h or 17–18 h post-transfection.

The same analysis was also conducted on neurons co-transfected with the CamKII-8 L-3’UTR and the MCP-mCherry plasmids to observe the behavior of the corresponding mRNA granules (Figure 1G). CamKII-8 L-3’UTR mRNA granules were visible in 26% of neurons already after 10–12 h, 34% of neurons after 17–18 h, 42% after 22–24 h, 68% after 36 h, and 97% after 68 h (Tukey’s multiple comparison test significance adjusted value of p 10–12 h vs. 17–18 h ns *p* > 0.9999; 10–12 h vs. 22-24 h ***p = 0.0002; 10–12 h vs. 36 h ****p ≤ 0.001). The number of CamKII-8 L-3’UTR mRNA granules detected (Figure 1H) increased from an average of 2.33 mRNA granules per neuron at 10–12 h to 5.3, 6.3, 8.3, and 9.11 mRNA granules per neuron at 17–18 h, 22–24 h, 36 h, and 68 h, respectively. CamKIIα mRNA granule diameter remained unchanged over time (Figure 1I, *n* = 3 independent cultures, *n* = 5–15 neurons for each culture). Considering the dynamics of these mRNA granules, the large majority of them resulted confined. Specifically, at 10–12 h, 100% mRNA granules were confined; at 17–18 h, mRNA granules were 81% confined, 5% anterograde, and 14% retrograde; at 22–24 h, 80% were confined, 13% anterograde, and 7% retrograde; at 36 h, 65% of the mRNA granules were confined, 20% anterograde, and 25% retrograde; and at 68 h, 55% were confined, 36% anterograde, and 9% retrograde (Figure 1J). The *χ*^2^ test showed a significantly different mRNA granule motility between 10 and 12 h and 17–18 h (*p* < 0.0001). Based on these results, the optimal time point to image neurons is 17–18 h post-transfection. This is because the number of CamkIIα mRNA granules is significantly higher (5 granules/neuron) compared to 10–12 h (2 granules/neuron, 10–12 h vs. 17–18 h, *p* < 0.0001), and no artefacts were observed due to MCP-mCherry alone. Additionally, the live imaging experiments suggest that the 17–18 h time point is suitable for dynamic analysis because CamkIIα mRNA granule movements can be observed in both anterograde and retrograde directions.

### 3.2 Tracking of BDNF mRNA trafficking in living neurons

Once the settings for visualising exogenous mRNA were established, hippocampal neurons were transfected with BDNF mRNA constructs and the MCP-mCherry at 11 DIV and living neurons were imaged on the following day, i.e., at 17–18 h post-transfection. The very same neuron was first imaged in a control solution (see Materials and Methods) for 10 min (min) to establish the baseline and then for 15 min in a solution containing 10 μM forskolin. To assess BDNF mRNA dynamics, we focussed on the BDNF transcript which has the most prominent dendritic localisation, i.e., with the 5’UTR encoded by BDNF exon 6 and the long version of the 3’UTR, and we compared it with the most somatically restricted BDNF transcript, i.e., the one with the 5’UTR encoded by BDNF exon 1, also having the long 3’UTR (Chiaruttini et al., 2009; Vicario et al., 2015; Colliva and Tongiorgi, 2021). In both BDNF transcripts, a 12 MS2 loop repeat was inserted at the end of the coding sequence (CDS) generating the constructs Ex6-bdnfCDS12L-3UTR, and Ex1-bdnfCDS-12 L-3UTR. As a positive control, the CamkIIα-8 L-3UTR construct was used, and as negative control a plasmid carrying only the 12 MS2 stem loop (12 L) was generated. This last plasmid has neither a coding sequence nor a 3’UTR sequence but just the same bacteriophages stem loops that were cloned in the BDNF and CamkIIα constructs.

**Figure 2:**
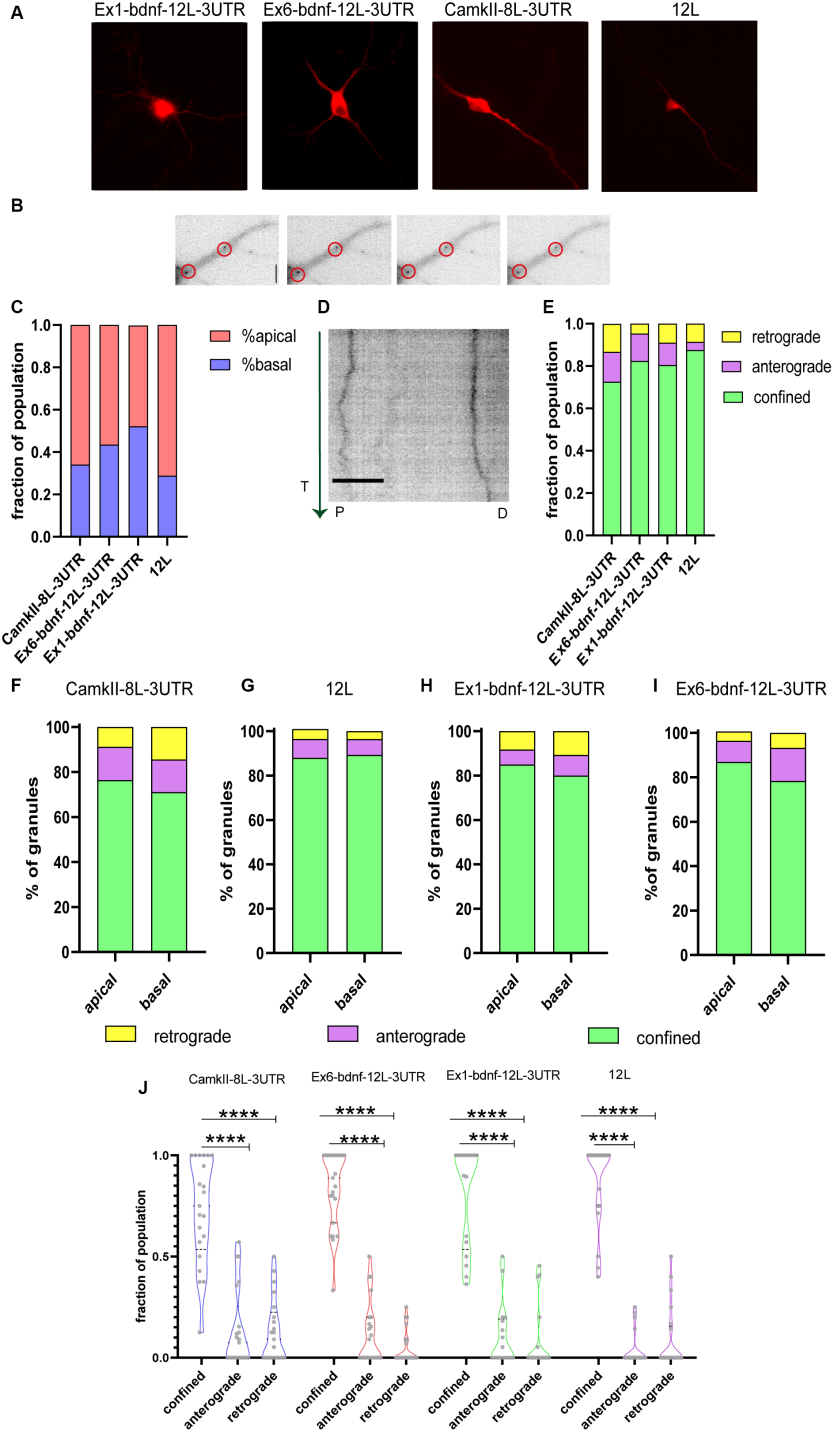
Tracking mRNA granules in live neurons. **(A)** Representative neurons transfected with the BDNF mRNA construct and MCP_mcherry, or CamkIIα and MCP_mcherry or the 12 L construct. **(B)** Representative dendrites with Ex6-bdnf-12 L-3UTR granules encircled. **(C)** Fraction of population of granules present at apical and basal dendrites. **(D)** Representative kymograph of a stretched dendrite (about 40 μm) showing the movements of two Ex6-bdnf-12 L-3UTR granules during the first 3 min of recording. The green vertical arrow on the left represents the time (T) direction from the beginning of the analysis (from 0 to 3 min). P and D represent the proximal and distal parts of the dendrite, respectively. Scale bar 10 μm. **(E)** Fraction of granule population showing the movement dynamic: the vast majority of granules show confined behavior (ANOVA followed by the Kruskal–Wallis post-hoc test). **(F)** Apical and basal granules of CamkIIα show a similar distribution of anterograde, retrograde, and confined granule behavior without any statically difference by the *χ*^2^ test, and the same test was applied for **(G)** 12 L apical and basal RNA granules and **(H)** Ex1-bdnf-12 L-3 UTR and **(I)** Ex6-bdnf-12 L-3UTR showing that apical and basal granules for each construct behave similarly without any significant statistical change. **(J)** Confined, anterograde, and retrograde fraction of population. The vast majority of granules were confined (ANOVA followed by the Kruskal–Wallis post-hoc test did not show a significant difference between the different constructs). Data shown were acquired from three independent experiments, and a total of 20 neurons per condition were imaged.

The dynamics of CamkIIα-8 L-3UTR, Ex6-bdnfCDS12L-3UTR, and Ex1-bdnfCDS-12 L-3UTR mRNA granule trafficking was evaluated along with the dynamics of the 12 L RNA (Figure 2A). Granules, whose typical appearance is shown for Ex6-bdnfCDS12L-3UTR in Figure 2B, were found in both apical and basal dendrites (Figure 2C) but with some differences in the percentage of localisation between these two compartments, depending on the mRNA analyzed. Specifically, 48% of granules showed an apical localisation and 52% basal for CamKII, 56% apical and 44% basal for Ex6, 66% apical and 34% basal for Ex1, and 71% apical and 29% basal for the 12 L.

Figure 2D illustrates a typical kymograph showing the behavior of two Ex6-bdnf-12 L-3UTR mRNA granules displaying little lateral movements during the observation time of 3 min (arrow on the left goes from to*p* = 0 min to bottom = 3 min) over a dendrite segment of 40 μm. The analysis of the kymographs obtained from both apical and basal dendrites showed that the large majority of the mRNA granules formed by the three constructs were confined, and also the control construct 12 L showed a very high percentage of confined RNA spots (Figure 2E), in analogy to previous reports (Doyle and Kiebler, 2011; Yoon and Gorospe, 2016). The graph in Figure 2E shows the fraction of mRNA granules displaying the three types of movements within the total granule population for each analyzed neuron. Regarding the active movements, the three constructs, but not the 12 L construct, displayed a slight bias, not statistically significant, for anterograde transport (fraction of population, anterograde vs. retrograde: CamKII: 0.075 upper limit 0.57 and lower limit 0 vs. 0.09 upper limit 0.5 and lower limit 0; Ex1-bdnf-12 L-3UTR: 0 upper limit 0.5 and lower limit 0 vs. 0 upper limit 0.25 and lower limit 0; Ex6-bdnf-12 L-3UTR: 0.5 upper limit 0 lower limit 0 vs. 0.45 upper limit 0 lower limit 0). For the construct 12 L, the anterograde and the retrograde transport showed similar results (median:0 upper limit 0.25 and lower limit 0 for the anterograde; 0 upper limit 0.5 and lower limit 0 for the retrograde). A statistical comparison for each granule class across the different constructs suggested no significant differences, except for a non-statistically significant trend for a higher confined population for the 12 L RNA granules with respect to CamkIIα (Figure 2E; *n* = 20–21 neurons for each construct, *p* = 0.13). Next, we verified whether the localisation of the granules in basal or apical dendrites could influence the type of movement they displayed (Figures 2F–I). The *χ*^2^ statistical test indicated that the percentage of movements showed by the mRNA granules was not significantly different between apical and basal dendrites, for all constructs (Figures 2F–I). As both basal and apical dendrites exhibited comparable percentages of confined, anterograde, and retrograde granules, the data from both compartments were combined for all subsequent analyses. This revealed that the majority of granules were confined (Figure 2J).

**Figure 3:**
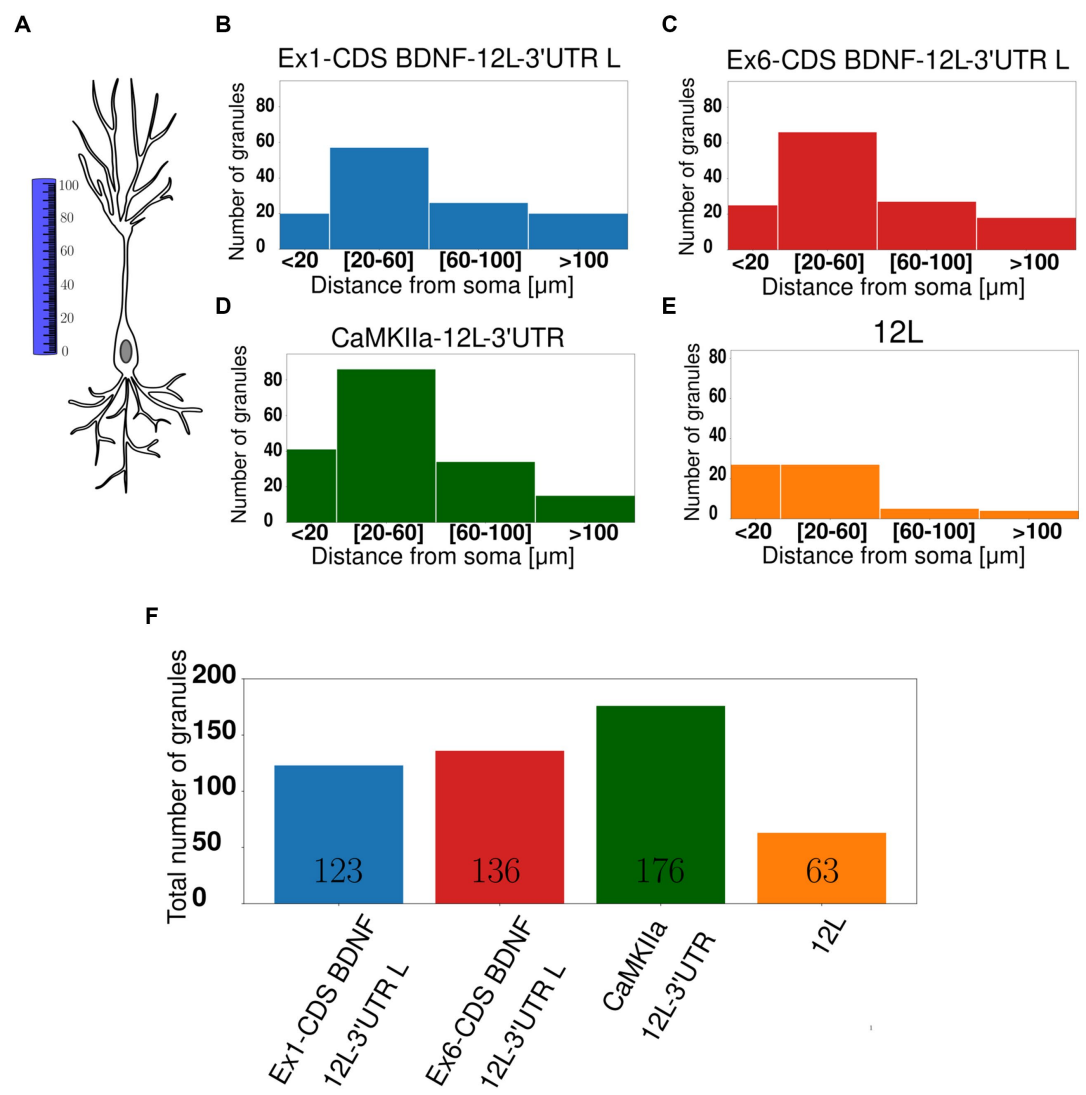
Subcellular localisation of mRNA granules. **(A)** Graphical representation showing how the distance from the soma was calculated. **(B)** Ex1-cdsBDNF-12 L-3UTR histogram with the number of granules detected at proximal (20–60 μm) and distal (60–100 μm) dendrites, and the majority of granules is at 20–60 μm. **(C)** Ex6-cdsBDNF-12 L-3UTR histogram. **(D)** CamkIIα-12 L-3UTR histogram even in this case the majority of granules is at proximal dendrites. **(E)** 12 L histogram. **(F)** Total number of granules detected in almost 20 neurons for each construct.

The distribution of the granules within the dendrites was also evaluated by counting the number of granules found in dendritic segments at increasing distance from the soma, namely, at <20 μm, between 20–6 μm, or 60–100 μm, and > 100 μm from the soma (Figure 3A). Histograms of granule distribution in neurons in resting conditions clearly show that the majority of granules for the four constructs was localized in the proximal dendrites, i.e., between 20 and 60 μm from the cell soma (Figures 3B–F).

### 3.3 Analysis of granule movements in resting neurons

**Figure 4:**
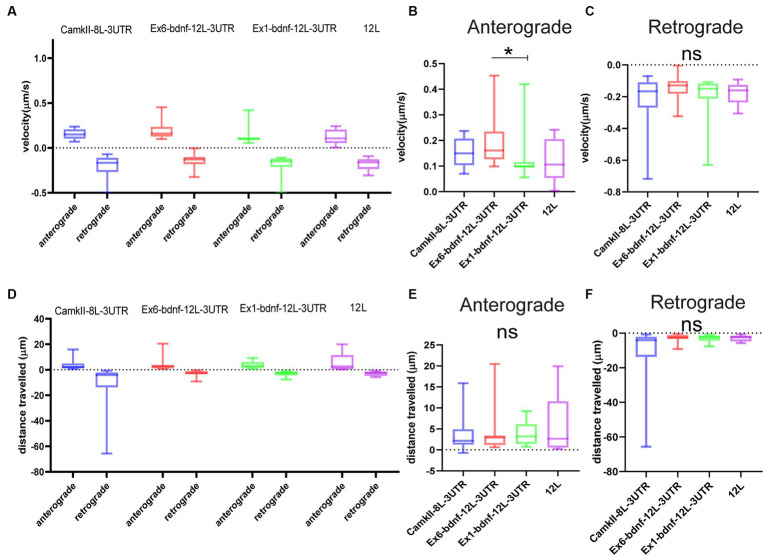
Type of movements of mRNA granules. **(A–C)** Anterograde and retrograde velocity for the four constructs. **(B)** Significant difference between Ex6 and Ex1 for anterograde velocity (*p* = 0.035) ANOVA followed by Kruskal–Wallis post-hoc test. **(D–F)** Total displacement of anterograde and retrograde movements. Data are represented by the median with 95% CI.

The population of granules with anterograde or retrograde active movements was analyzed in resting neurons for their velocity and the distance traveled during the observation time. The four constructs showed similar velocities (Figures 4A–C). For the anterograde movements (Figure 4B), velocities were CamKII: 0.153 μm/s, Ex1-bdnf-12 L-3UTR: 0.133 μm/s, Ex6- bdnf-12 L-3UTR: 0.189 μm/s, and 12 L: 0.125 μm/s. For the retrograde movements, velocities were CamKII: 0.234 μm/s, Ex1-bdnf-12 L-3UTR: 0.195 μm/s, Ex6-bdnf-12 L-3UTR: 0.143 μm/s, and 12 L: 0.176 μm/s (Figure 4C). The velocities observed are consistent with values previously reported in the literature (Chiaruttini et al., 2009), and the only statistically significant difference was found between Ex6-bdnf-12 L-3UTR and Ex1-bdnf-12 L-3UTR (*p* = 0.035), with the Ex6 construct showing a higher anterograde velocity than the Ex1 construct (Figure 4B). In contrast, no significant differences were found among the different constructs for the retrograde transport (Figure 4C). The distance traveled during the observation period was similar for all mRNA constructs (Figure 4D). The anterograde movement traveled distance for CamKII was 3.5 μm, for Ex1-bdnf-12 L-3UTR was 3.9 μm, for Ex6-bdnf-12L3UTR was 3.4 μm, and for 12 L was 5.3 μm (Figure 4E). The retrograde movement distance for CamKII was −10.790 μm, for Ex1-bdnf-12 L-3UTR was −2.9 μm, for Ex6-bdnf-12L3UTR was −3.1 μm, and for 12 L was −2.8 μm (Figure 4F).

### 3.4 Analysis of granule movements after cLTP induction

**Figure 5:**
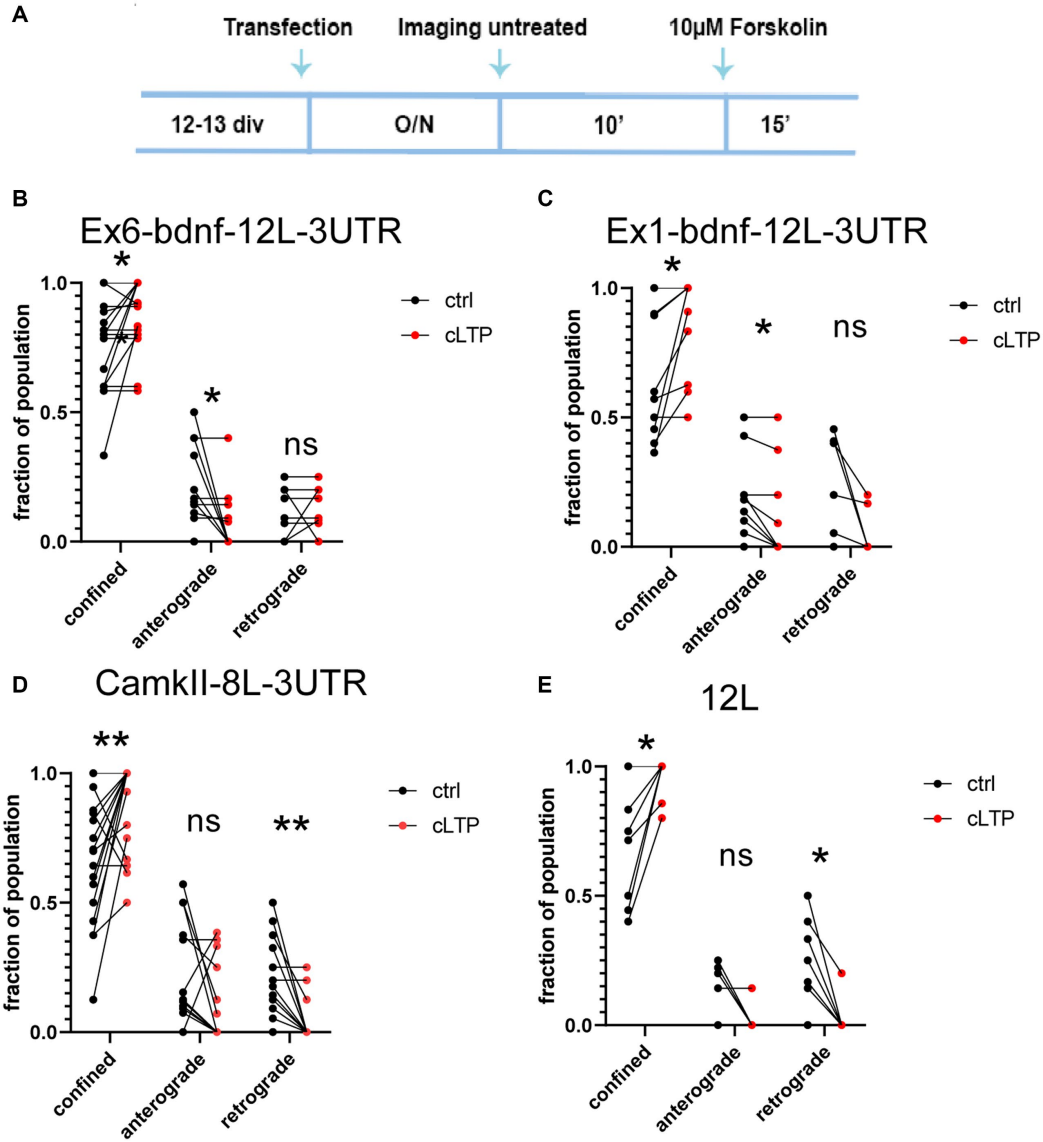
Dynamics of mRNA granules after cLTP induction. **(A)** Experimental outline: after an overnight (O/*N* = 17-18 h) from transfection, neurons were imaged for 10′ in a control medium (untreated), and then, the same neurons were incubated with 10 μM Forskolin and imaged for an additional 15 min. **(B)** After cLTP, Ex6-bdnf-12 L-3UTR granules showed an increased fraction of confined granules (*p* = 0.0234) and a decrease in the fraction of anterograde granules (*p* = 0.0156). **(C)** After cLTP, Ex1-bdnf-12 L-3UTR showed an increase in the confined granule population (*p* = 0.0156) and a decrease in the anterograde granules (*p* = 0.0313). **(D)** CamkIIα-8 L-3UTR showed an increase in the confined granules (*p* = 0.0084) and a decrease in the retrograde granules (*p* = 0.0020). **(E)** 12 L showed an increase in the confined granule population (*p* = 0.0313) and a decrease in the retrograde granules (*p* = 0.12). Statistical analysis was performed by paired *t*-test.

To investigate the impact of plasticity on BDNF mRNA granule dynamics, we utilized a chemical protocol that induces long-term potentiation (cLTP) through the use of 10 μM forskolin. We confirmed the effectiveness of the potentiation by quantifying the nuclear immunofluorescence for c-fos, an immediate early gene commonly used to assess neural activity (Bullitt, 1990; Ferhat et al., 1993; Herdegen and Leah, 1998) (Supplementary Figure S1A). The c-fos mean fluorescence intensity was evaluated using a custom MACRO created as described in the Materials and Methods section. The results from *n* = 4 independent experiments (between 900 and 3,000 cells analyzed for each condition) showed that the c-fos protein expression in the nucleus was significantly higher after 90 min (ctrl vs. cLTP *p* = 0.037) but not at 30 min (ctrl vs. cLTP *p* = 0.0649) or 60 min (Supplementary Figure S1B). In addition, we evaluated the c-fos intensity of cultures that were treated with 10, 20, or 50 mM KCl for 30, 60, and 90 min (Supplementary Figures S1C,D). Cumulative plots indicated that 90 min is the time at which the population of neurons showed the highest c-fos fluorescence intensity following KCl-induced depolarisation (Supplementary Figures S1C,D). After 10 min of baseline imaging, 10 μM forskolin was added to chemically induce LTP and each neuron was imaged for an additional 15 min (Figure 5A). Following cLTP induction, granules exhibited a movement arrest and showed confined behavior (Figures 5B–E; *n* = 20–21 neurons). A paired *t*-test statistical analysis showed that, comparing the behavior of the same granules in the ctrl and cLTP condition, the confined population was significantly increased after cLTP for Ex6 (*p* = 0.0234, panel 5B), Ex1 (*p* = 0.0156, panel 5C), CamkIIα (*p* = 0.0084, panel 5D), and 12 L (*p* = 0.0156, panel 5E). For the anterograde population, there was a significant decrease after cLTP with respect to ctrl, for Ex6 (*p* = 0.0156, panel B) and Ex1 (*p* = 0.0313, panel 5C), but neither for CamkIIα (*p* = 0.11, panel 5D) nor for 12 L (*p* = 0.12, panel 5E). After the cLTP induction, the retrograde population was significantly reduced for CamkIIα (*p* = 0.0020, panel 5D) and 12 L (*p* = 0.0313, panel 5E). The finding that the 12 L RNA granules exhibited a behavior comparable to that of the CamkIIα mRNA was unexpected because the 12 L construct was not predicted to have any biological significance.

### 3.5 cLTP-induced confinement of BDNF mRNA in proximity to a spine

**Figure 6:**
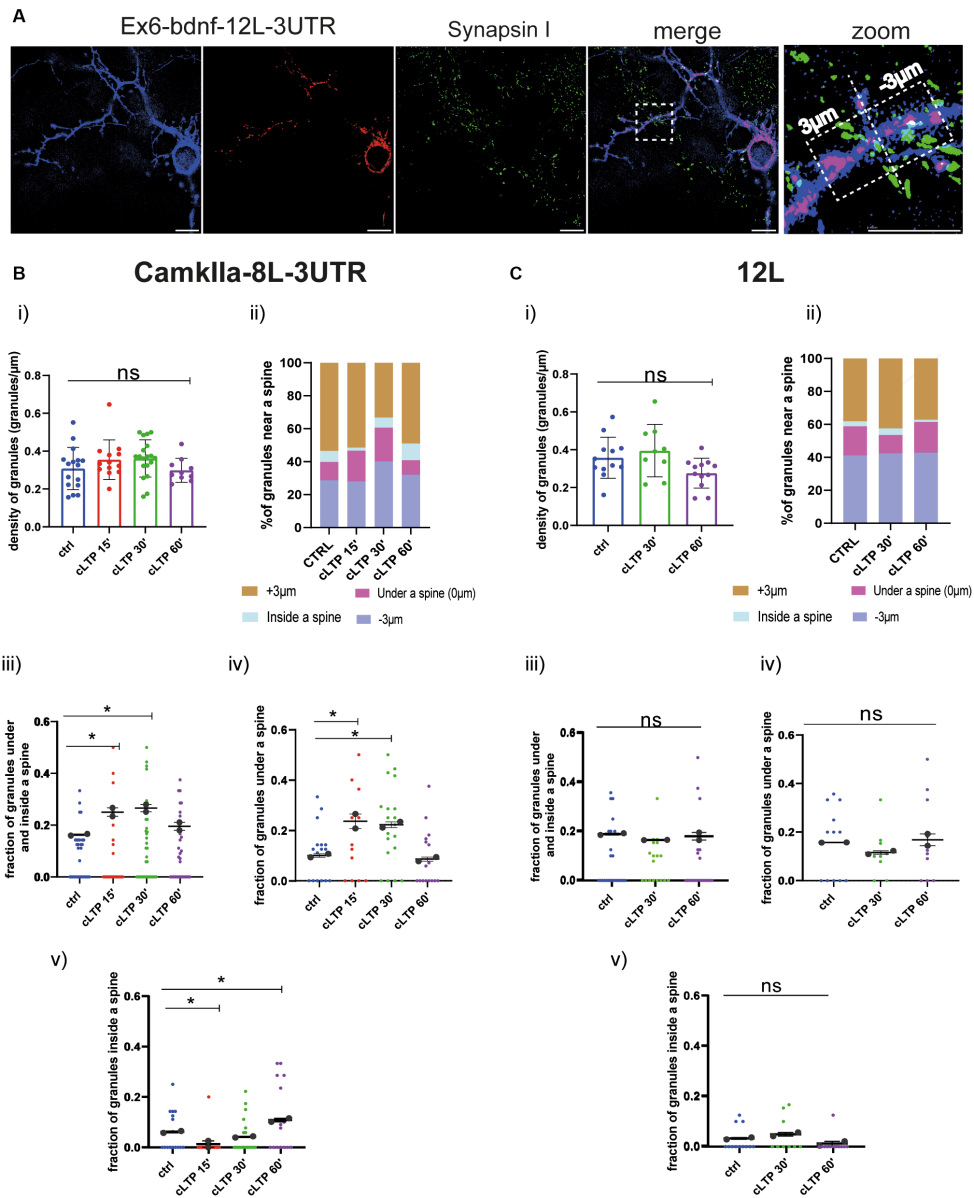
CamkIIα mRNA and 12 L RNA granules dynamics in proximity to dendritic spines. **(A)** Representative thresholded images of Ex6-BDNF-12 L-3UTR and Synapsin I. From the high magnification section, a spine is visible, and granules found in the range of +3 μm and − 3 μm were considered. Scale bar 5 μm. **(B)** CamkIIα-8 L-3UTR granules. (i) CamkIIα mRNA granules counted in each dendrite stretch of 30–50 μm. ANOVA followed by Dunnett’s *post-hoc* test revealed no significant difference in the density of mRNA CamkIIα granules after cLTP stimulation. (ii) mRNA granule distribution near a spine after cLTP induction. Granules under a spine and inside a spine were considered. (iii) CamkIIα granules under and inside a spine were considered at different cLTP induction. After 15 min and 30 min from cLTP induction, there is an increase in the granules present under and inside a spine (ctrl vs. cLTP 15′ *p* = 0.0311, ctrl vs. cLTP 30′ *p* = 0.0180). (iv) CamkIIα granules under a spine were significantly higher after 15 min and 30 min from cLTP induction (*p* = ctrl vs. cLTP 15′ *p* = 0.0140 and ctrl vs. cLTP 30′ *p* = 0.0204). (v) CamkIIα granules inside a spine were significantly higher after 60 min from cLTP ctrl vs. 15′ *p* = 0.03369 and ctrl vs. cLTP 60′ *p* = 0.00336. **(C)** 12 L RNA granules. (i) 12 L RNA granules counted in each dendrite stretch of 30–50 μm ANOVA followed by Dunnett’s post-hoc test revealed no significant difference in the density of 12 L granules after cLTP stimulation. (ii) RNA granule distribution near a spine after cLTP induction. (iii) 12 L RNA granules under and inside a spine were considered at different cLTP induction. (iv) 12 L RNA granules under a spine were not significantly higher after cLTP induction. (v) 12 L RNA granules inside a spine were not significantly higher after cLTP.

Based on the live-cell analysis, we concluded that mRNA granules become stationary after cLTP stimulation as they arrest their anterograde movements. However, due to resolution limitations, it was unclear whether after the cLTP-induced stop of mRNA trafficking, granules accumulated in the proximity of a spine or not. To clarify this point, neurons transfected with the BDNF constructs Ex6-bdnfCDS-12 L-3UTR, Ex1-bdnfCDS-12L3UTR and the control constructs CamkIIα-8 L-3’UTR and 12 L were analyzed with the super-resolution Elyra7 SIM microscope. Neurons were fixed at different time points, i.e., at 15 min, 30 min, and 60 min from cLTP induction. Then, neurons were immunolabeled with Synapsin 1 to identify the position of presynaptic terminals, and the images were acquired (see Materials and Methods). During post-processing analysis, thresholded images of the mRNA granules and Synapsin 1 were created (Figure 6A, see also Supplementary Figure S2). Spines were identified primarily by the shape outlined by the overall neuronal fluorescence (post-colored in blue), i.e., where there was a protrusion of the right shape and size to be a spine. Second, we looked specifically at Synapsin 1-labeled protrusions along the dendrites. Since only a few neurons were co-transfected with the mCherry filler and MS2 reporter, many green Synapsin I spots belong to presynaptic terminals that impinge on the postsynaptic compartments of unstained neurons. To identify mushroom and stubby spines, the head diameter and the neck of each spine were measured. Both spine types were considered for the analysis. For each dendrite, a ROI of 30–50 μm was drawn, and all the spines in it were considered. The density of granules was counted in each ROI (granules/μm = number of granules per length unit), and the number of granules located within +3 μm (granules more distant to the soma) and − 3 μm (granules closer to the soma) from each spine was considered. For this purpose, an orthogonal axis dividing the spine into two halves was designed as a reference line (see Figure 6A). Granules were also classified as being inside the spine head or in the dendritic shaft segment below the spine. In both cases (inside or below the spine), a position closer to 0 indicates that the granule is positioned close to the spine axis which is generally running in correspondence to the spine neck (see Figure 6A).

The density of granules for CamkIIα and 12 L counted in each dendrite stretch of 30–50 μm did not change at the different time points analyzed after cLTP induction (Panel (i) in Figures 6B,C for CamkIIα and 12 L, respectively). However, when considering the CamkIIα granules located under the spine and those found inside the spine as a single fraction of the whole population, a significant increase was found at 15 min and 30 min after cLTP induction, with respect to baseline (Figures 6B,C Panel iii, ctrl vs. cLTP 15′ *p* = 0.0311, ctrl vs. cLTP 30′ *p* = 0.0180; summarized as % in Panel ii). Interestingly, when the two fractions of CamkIIα granules located under and inside the spine were considered separately, an opposite behavior was found. In detail, the fraction of granules under the spine transiently increased at 15 min and 30 min to return to baseline levels at 60 min after cLTP (Figure 6B, Panel iv, ctrl vs. cLTP 15′ *p* = 0.0140, and ctrl vs. cLTP 30′ *p* = 0.0204; summarized as % in Panel ii), while the fraction of CamkIIα granules located inside a spine was significantly decreased in the early phase after cLTP (15 min), to become significantly higher than the baseline in the late phase, i.e., at 60 min post-cLTP (Figure 6B, Panel v, ctrl vs. 15′ *p* = 0.03369 and ctrl vs. cLTP 60′ *p* = 0.00336; summarized as % in Panel ii). As expected, the negative control 12 L RNA granules did not distribute differently inside and under a spine neither in control conditions nor after cLTP stimulation (Figure 6C, Panels i–v).

**Figure 7:**
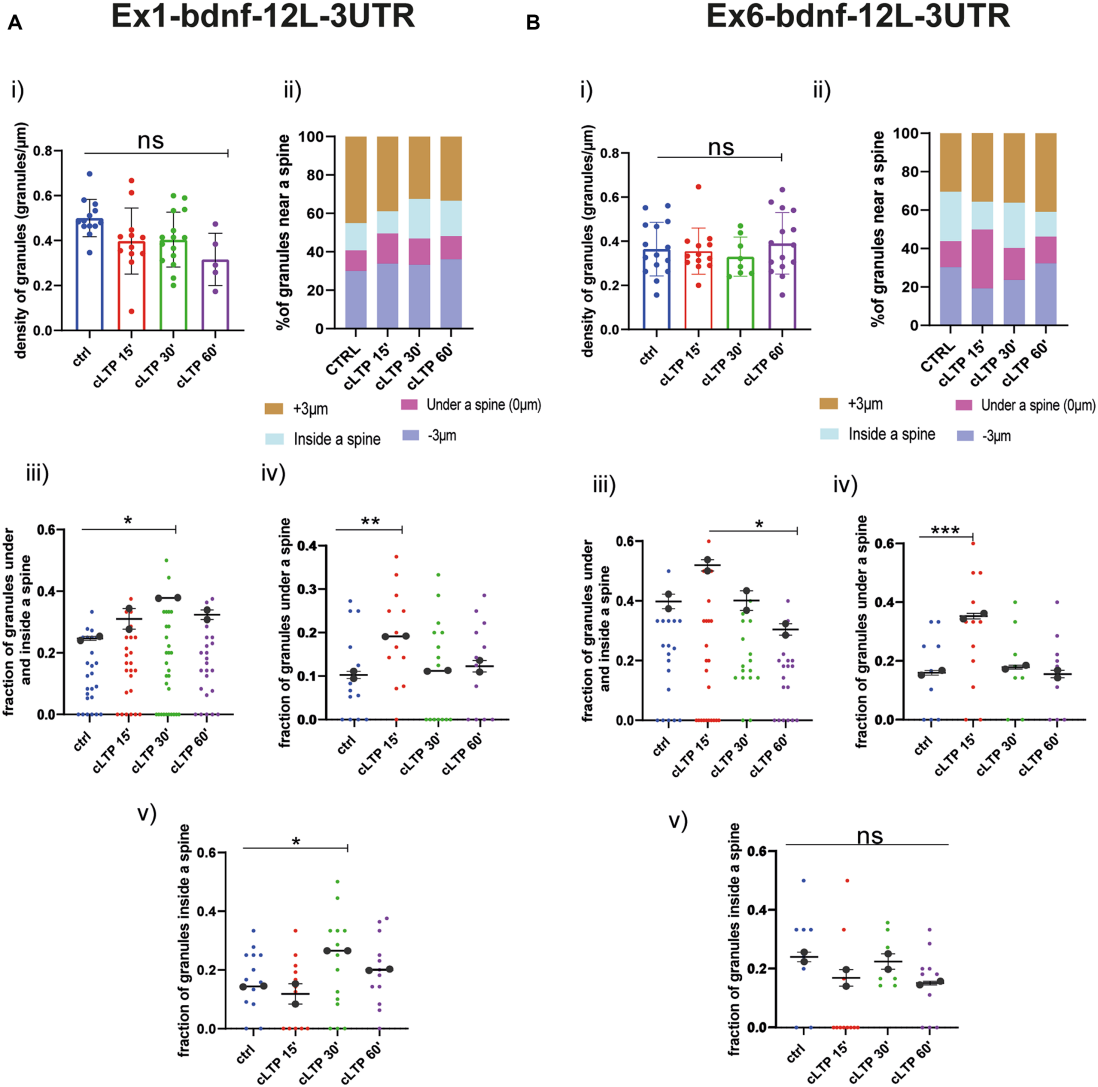
BDNF mRNA granules trafficking near a spine. **(A)** Ex1-bdnf-12 L-3UTR. (i) mRNA granules counted in each dendrite stretch of 30–50 μm ANOVA followed by Dunnett’s post-hoc test revealed no significant difference in the density of Ex1-bdnf mRNA granules after cLTP stimulation. (ii) mRNA granule distribution near a spine after cLTP induction. Granules under a spine and inside a spine were considered. (iii) Ex1bdnf granules under and inside a spine were considered at different cLTP induction. After 30 min from cLTP induction, there is an increase in the granules present under and inside a spine. (iv) Ex1bdnf granules under a spine were significantly higher after 15 min from cLTP induction ctrl vs. cLTP 15′ *p* = 0.047. (v) Ex1bdnf granules inside a spine were significantly higher after 30 min from cLTP ctrl vs. 30′ *p* = 0.0253. **(B)** Ex6-bdnf-12 L-3UTR. (i) mRNA granules counted in each dendrite stretch of 30–50 μm ANOVA followed by Dunnett’s post-hoc test revealed no significant difference in the density of mRNA CamkIIα granules after cLTP stimulation. (ii) mRNA granule distribution near a spine after cLTP induction. Granules under a spine and inside a spine were considered. (iii) Ex6bdnf granules under and inside a spine were considered at different times after cLTP induction. There is a significant increase between 15 min and 60 min from cLTP induction. (iv) Ex6bdnf granules under a spine were significantly higher after 15 min from cLTP induction (*p* = ctrl vs. 15’ cLTP *p* = 0.0005). (v) Ex6bdnf granules inside a spine were not significantly higher after any time from cLTP. Statistical analysis was done by ANOVA followed by Tukey’s *post-hoc* test.

Regarding the BDNF constructs, also for Ex1-bdnfCDS-12 L-3UTR (BDNF-Ex1) and Ex6-bdnfCDS-12 L-3UTR (BDNF-Ex6), there was no statistically significant change, with respect to baseline, in the granule’s density at the various time points after cLTP (Figure 7A for BDNF-Ex1 and Figure 7B for BDNF-Ex6, Panels i and ii). A significant increment in the pooled fraction of granules inside and under a spine was observed after 30 min from cLTP induction (ctrl vs. cLTP 30′ *p* = 0.0266) but not at 15 min or 60 min (ctrl vs. 15′ *p* = 0.22 and ctrl vs. 60′ *p* = 0.13). When considering the granules inside and under the spine separately, there was an increase in BDNF-Ex1 mRNA granules under a spine after 15 min from cLTP (Figure 7A, panel iv; ctrl vs. cLTP 15′ *p* = 0.0047) and an increase in BDNF-Ex1 mRNA granules inside the spine after 30 min from cLTP (ctrl vs. 30′ *p* = 0.0253; Figure 7A, panel v). For the BDNF-Ex6 construct, the pooled fraction of granules inside and below a spine was significantly higher at 15 min with respect to 60 min (*p* = 0.0115) but not with respect to the control condition (*p* = 0.0785; Figure 7B, panel iii). Examining these granule populations separately, we observed an increase in the fraction of granules located under a spine after 15 min from cLTP induction (Figure 7B, panel iv; ctrl vs. 15’ cLTP *p* = 0.0005). However, the fraction of granules inside the spine remained unchanged (Figure 7B, panel v, ctrl vs. cLTP 60′ *p* = 0.1271). Taken together, these results indicate that the distribution of BDNF mRNA within the dendrites changes after cLTP induction. Specifically, there is a significant accumulation of BDNF mRNA granules under the spine in the early phase after cLTP induction, i.e., at 15 min, followed by a translocation of at least the BDNF-Ex1 mRNA variant inside the spine at 30 min and a return to baseline at 60 min from cLTP stimulation.

### 3.6 Basal trafficking and distribution of BDNF protein in living neurons

**Figure 8:**
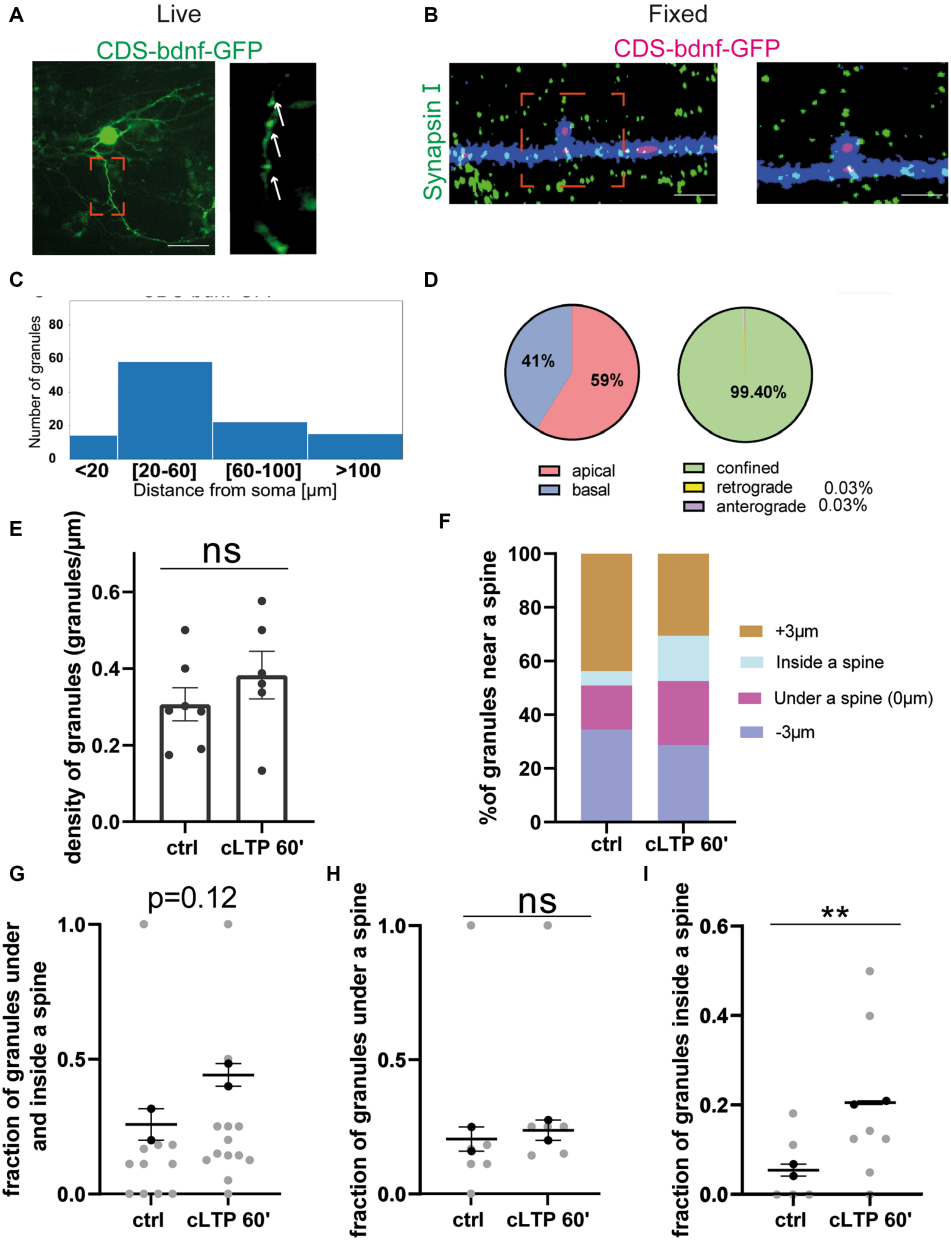
BDNF protein near a spine: **(A,B)** Representative images of CDS-bdnf-GFP spots transfected neurons. **(A)** Living neurons transfected with CDS-bdnf-GFP. **(B)** Neurons were fixed and labeled with Synapsin I. Scale bar 2 μm. **(C)** Histogram of the CDS-bdnf-GFP protein spot distribution in the different dendritic compartments. **(D)** Percentage of CDS-bdnf-GFP spots granules at apical and basal dendrites (left) and the percentage of spots confined, anterograde, and retrograde (right). Almost all the protein spots resulted to be confined. **(E)** CDS-bdnf-GFP spots on the left, protein spots counted in each dendrite stretch of 30–50 μm. An unpaired *t*-test revealed no significant difference in the density of CDS-BDNF spots after cLTP stimulation. **(F)** Protein spot distribution near a spine after cLTP induction. Spots under a spine and inside a spine were considered. **(G)** Protein spots under and inside a spine were considered at different cLTP induction. After 60 min from cLTP induction, there is a tendency for BDNF protein spots to increase under and inside a spine (*p* = 0.12). **(H)** CDS-BDNF granules under a spine were not significantly higher after cLTP induction. **(I)** CDS-BDNF spots inside a spine were significantly higher after 60 min from cLTP ctrl vs. 60′ *p* = 0.0085.

After having established that cLTP induces a trafficking arrest of BDNF mRNA granules and redistributes them to accumulate under and partly inside the spines, it remained to determine how the BDNF protein is redistributed during LTP. Accordingly, in the following series of experiments, the dynamic movements of the BDNF protein were recorded in living neurons because of one chimeric construct consisting in the BDNF coding sequence (CDS) common to all BDNF transcript variants fused to the green-fluorescent protein (GFP). Previous studies have shown that the mRNA from this construct, named CDS-BDNF-GFP, has a dendritic localisation and produces a protein that is secreted from postsynaptic terminals (Chiaruttini et al., 2009; Baj et al., 2011; Brigadski and Leßmann, 2020). Neurons were co-transfected at DIV 9–10 using the CDS-BDNF-GFP construct together with mCherry as a filler and were imaged 2 days later, as previously reported (Baj et al., 2011). As shown in Figures 8A,B, neurons transfected with CDS-BDNF-GFP showed clearly identifiable fluorescent protein spots in dendrites (Figure 8A) and within one spine (Figure 8B). The histogram shown in Figure 8C presents the distribution of CDS-BDNF-GFP spots in the dendritic compartment at increasing distance from the soma of neurons cultured in basal conditions. Specifically, we found 15 spots within a distance of less than 20 μm from the cell soma, 60 spots within a distance of 20–60 μm, 25 spots within a distance of 60–100 μm, and 15 spots farther than 100 μm from the cell soma (*n* = 21 neurons; *n* = 3 independent cultures). In untreated cultures, CDS-BDNF-GFP protein spots were detected in both apical (59%) and basal dendrites (41%) (Figure 8D, left), and the confined population represented almost the totality of all GFP-tagged BDNF protein spots (99.40%, Figure 8D, right).

### 3.7 cLTP-induced redistribution of the BDNF protein in proximity to a spine

The same analysis performed to study the effects of cLTP on mRNA granule distribution and motility was carried out also for the BDNF protein. Using the fluorescent CDS-BDNF-GFP construct, we found that the density of BDNF protein was not changed after 60 min from the induction of cLTP (Figure 8E, ctrl vs. cLTP 60 min *p* = 0.12; *n* = 8–10 neurons). Of note, during the live imaging, we detected newborn spots with a frequency of 8.64% of the total spot population. However, these events seemed to not significantly affect the density of protein spots after 60 min from cLTP induction, possibly due to either a physiological secretion or normal turnover of the protein. The analysis of the distribution of the BDNF protein spots near, under, or within a spine (Figures 8F,H *n* = 15–20 neurons) revealed a significant increment of BDNF protein spots inside a spine at 60 min after cLTP induction (Figure 8I etc) (ctrl vs. cLTP 60′ *p* = 0.0085). Taken together, these results suggest that in the late phase after cLTP induction (1 h), BDNF becomes more present inside dendritic spines.

## 4 Discussion

The primary biological question behind this study pertains to the dynamics of BDNF mRNA trafficking and its local translation at synapses in response to synaptic plasticity. Our study suggests that the early phase following LTP induction (i.e., 15 min) is characterized by a general arrest of dendritic mRNA trafficking, along with an increase in the local density of mRNA granules beneath, but not within, the spines. The behavior of the control CaMKIIα mRNA and exon-1 and exon-6 BDNF mRNA was similar in the early-LTP phase, with an increase in the number of mRNA granules under the spine at 15 min and 30 min. However, in the late phase of LTP (i.e., 60 min), CaMKIIα mRNA increased inside the spine, whereas BDNF mRNA did not. At the protein level, we found that newly synthesized BDNF was accumulated inside the spine at 60 min after cLTP induction. Taken together, these results suggest that LTP triggers a process involving protein translation at the basis of the spine from locally confined mRNAs, followed by translocation of the protein (or more likely, the vesicles containing it) inside the spines, toward the sites of release at synapses.

The first goal of this study was to set up the conditions to study the dynamics of BDNF mRNA and protein trafficking in living neurons. The tool chosen for the visualization of BDNF mRNA was the MS2 RNA loops, consisting of 12 bacteriophage loops cloned to our cDNA of interest (ex1-cdsBDNF-3 UTR and ex6-cdsBDNF-3UTR). This is a well-established technique that has been widely used before (Tutucci et al., 2018; Holt et al., 2019). Although the actual number of MS2 loops varies from 6 to 120 in the various previous studies (Tutucci et al., 2018; Bauer et al., 2019), we found that 12 loops were sufficient to provide a strong signal that could be reliably recorded in live imaging analysis within a reasonable dynamic range after compensating for the natural bleaching of the signal. The sequence of the 12 RNA loops is derived from the bacteriophage MS2 and is supposed to have no similarity to any other mammalian RNA sequence. Accordingly, it is not expected to have any biological functions in neurons. However, recent findings have challenged this notion. It has been observed that when the MS2 loop is bound to the MS2 coat protein, it may interfere with the mRNA decay in the yeast *Saccharomyces cerevisiae* and lead to the formation of 3′ decay fragments containing MS2 arrays (Tutucci et al., 2018). Interestingly, in this study, the accumulation of mRNA fragments was more pronounced for short-lived mRNAs, while for constitutively expressed and stable mRNAs the phenomenon was less significant because fewer molecules are degrading at any one time. Of note, considering 20 neurons imaged for each construct, the total number of granules detected for the 12 L was significantly lower than that detected for the other constructs. In addition, the RNA granules formed by the 12 L construct showed more proximal localisation in comparison with all other constructs used. In conclusion, the use of the “classical” MS2 system for BDNF mRNA is acceptable.

For the protein visualization, chimeric BDNF-GFP constructs have been used. In addition, this tool is very well established for BDNF analysis (Baj et al., 2011; Brigadski and Leßmann, 2020) likely because GFP protein dimerisation, a major problem in the use of GFP as a reporter protein, is not relevant in a molecule such as BDNF, which is a dimer by itself. Of note, to obtain a more natural behavior of the mRNA, the two BDNF exon1 and exon6 isoforms used here included the 3’UTR Long tail which is considered more abundant in dendritically targeted BDNF mRNAs (Vicario et al., 2015).

One of the main findings of this study is that the vast majority of granules of the four constructs exhibited a confined behavior (EX1-cds BDNF-12 L-3’UTR, EX6-cds BDNF-12 L-3’UTR, CamkIIα-8 L-3’UTR and 12 L). This result is in agreement with a recent study (Donlin-asp et al., 2021) that employed molecular beacons to track endogenous CamkIIα and PSD95 mRNAs. In analogy with that study, we also observed that the vast majority of granules exhibiting an anterograde or retrograde movement ceased to move and remained restricted following cLTP induction. The validity of our approach, which uses an exogenous system such as the MS2 12-loop technique, was thus confirmed by the comparison with studies that had the same findings by investigating the dynamics of endogenous mRNAs (Donlin-Asp et al., 2021).

Intriguingly, the EX1-cds BDNF-12 L-3’UTR, EX6-cds BDNF12L-3’UTR, and CamkIIα-8 L-3’UTR showed a slight bias, even though not significant, for anterograde movements. This bias could be caused by the 3’UTR sequence that, as already reported, is important for the dendritic localisation of BDNF mRNA (Vicario et al., 2015) and other mRNAs (Bauer et al., 2019). This explanation is strengthened by the observation that the 12 L construct, without the 3’ UTR sequence, did not show any bias for the anterograde movements, showing an equal distribution of anterograde and retrograde movements. The anterograde velocity for the EX6-cds-BDNF-12 L-3’UTR showed a significant increment when compared with the anterograde velocity for the EX1-cds BDNF-12 L-3’UTR. This can be linked to a previously reported finding that the Ex6-BDNF is located in more distal parts of dendrites in comparison with the Ex1-BDNF (Baj et al., 2011). Another intriguing result is related to the bias for the localisation in apical dendrites found for BDNF mRNAs, while for CaMKIIα the distribution between apical and basal dendrites was almost equal. In hippocampal neurons, apical and basal dendrites have very different morphology and distinct functions as well as intrinsic and extrinsic connections with most excitatory synapses being located on apical dendrites of pyramidal neurons. The preference for apical dendrites of exon 6 BDNF isoform which is one of the most germane dendritic BDNF mRNA isoforms strengthens our hypothesis that this BDNF transcript is the preferential source of dendritic BDNF protein at excitatory synapses.

Previous studies showed that Ex1 BDNF transcript was mainly located at proximal dendrites, whereas Ex6 BDNF mRNA has been identified beyond 100 μm from the cell soma (Chiaruttini et al., 2009; Baj et al., 2011; Mallei et al., 2015). Surprisingly, upon stimulation with Forskolin, the EX6-bdnfCDS-12L3UTR could not be detected in the most distal dendritic compartment. This could probably be due to the timing and nature of the stimulus. Indeed, using a similar construct with the 12 L substituted for GFP (EX6-bdnfCDS-GFP-3UTR), a very distal localisation was obtained after 3 h of stimulation with 10 mM KCl (Baj et al., 2011), whereas in this study 15 min of 10 μM Forskolin may not have been sufficient for BDNF mRNA to reach the distal compartment of dendrites.

One further goal of this study was to understand whether after cLTP induction, BDNF mRNA is locally accumulated and eventually translated into protein in proximity to the spines or whether mRNA and protein have a random distribution along the dendrites. Using super-resolution microscopy, we measured the distance between mRNA granules and the nearest spine. Additionally, we investigated whether the granules were located either beneath or inside the spine. Although with this analysis it was not possible to specifically identify the potentiated spines, the results highlighted that in the early-LTP phase, both CamkIIα and BDNF granules were generally increased under the spines, while in the late-LTP phase only CamkIIα granules were accumulated inside the spine. A possible interpretation is that after short periods (15–30 min) from cLTP induction, a rise in Ca^2+^ concentrations in the dendritic shaft underneath activated spines provokes an uncoupling of the mRNA granules from the kinesins/microtubule-driven transport leading the mRNAs to become stationary near the activated spines. While for BDNF mRNA granules, the dendritic segment under the spine is the final destination, likely due to the need for this secreted protein to be processed through the secretory pathway, CamkIIα mRNA which encodes for a cytoplasmatic protein may be further recruited by an actin-based transport system which drives this mRNA into the spines where it becomes locally translated (Hanus and Ehlers, 2008). This model is compatible, with the finding that after 60 min from cLTP induction, we observed an increment of BDNF protein spots inside a spine. Of note, the construct with only the 12 stem loops (12 L) did not show any significant increase in the number of granules under or inside the spine, confirming that the distribution of the 12 L granules is cLTP-independent.

Given the critical role L-LTP played local BDNF synthesis, a dendritic localisation of the translational machinery for secretory proteins is required. In addition, BDNF undergoes a number of post-translational modifications that involve the secretion pathway including proper folding, glycosylation, cleavage, and sorting to the constitutive or regulated secretory route. Folding and N-glycosylation are processed in ER, whereas cleavage and sorting occur in the Golgi apparatus. In the past years, Ehlers et al. have shown that at least in cultured hippocampal neurons, a complete Golgi apparatus is present only in a minority of dendrites and is located near the cell soma (Horton and Ehlers, 2003; Wang et al., 2012). However, it has been shown that small Golgi-like organelles (so-called Golgi outposts) are selectively localized at dendritic branch points and in distal dendrites. Thus, all the secretory pathway elements are available in dendrites. In a previous study using electron microscopy *in situ* hybridisation, we showed that BDNF mRNA is detected in association with polyribosomes located under spines and near dendritic branches, suggesting active translation without interaction with the endoplasmic reticulum (Tongiorgi et al., 2004). At present, how secretory or transmembrane proteins synthesized locally on polyribosomes can be subsequently processed via the secretory pathway remains a conceptually challenging issue.

## Data availability statement

The raw data supporting the conclusions of this article will be made available by the authors, without undue reservation.

## Ethics statement

The animal study was approved by the Organismo Preposto al Benessere degli Animali (OPBA) of the University of Trieste and by the Italian Ministry of Health. The study was conducted in accordance with the local legislation and institutional requirements.

## Author contributions

GB: Data curation, Formal analysis, Investigation, Writing – original draft. ET: Conceptualization, Funding acquisition, Project administration, Supervision, Writing – review & editing.
