# Supplementary material for: Chemical LTP induces confinement of BDNF mRNA under dendritic spines and BDNF protein accumulation inside the spines
Source: Front Mol Neurosci. 2024 Feb 21;17:1348445. doi: 10.3389/fnmol.2024.1348445 (PMC10914971; doi:10.3389/fnmol.2024.1348445)
Supplement: Supplementary file 1 [file Data_Sheet_1.PDF]

Supplementary Material

Chemical-LTP induces confinement of BDNF mRNA under dendritic spines and BDNF protein accumulation inside the spines

Giorgia Bimbi[1]; Enrico Tongiorgi[1]*

Department of Life Sciences, University of Trieste, 34127 Trieste, Italy

**Corresponding author:*

Department of Life Sciences, University of Trieste,

Via Licio Giorgieri, 5 - 34127 Trieste, Italy

tongi@units.it

Running title: ***cLTP-induced localization of BDNF in spines***

1 Supplementary Figures and Tables

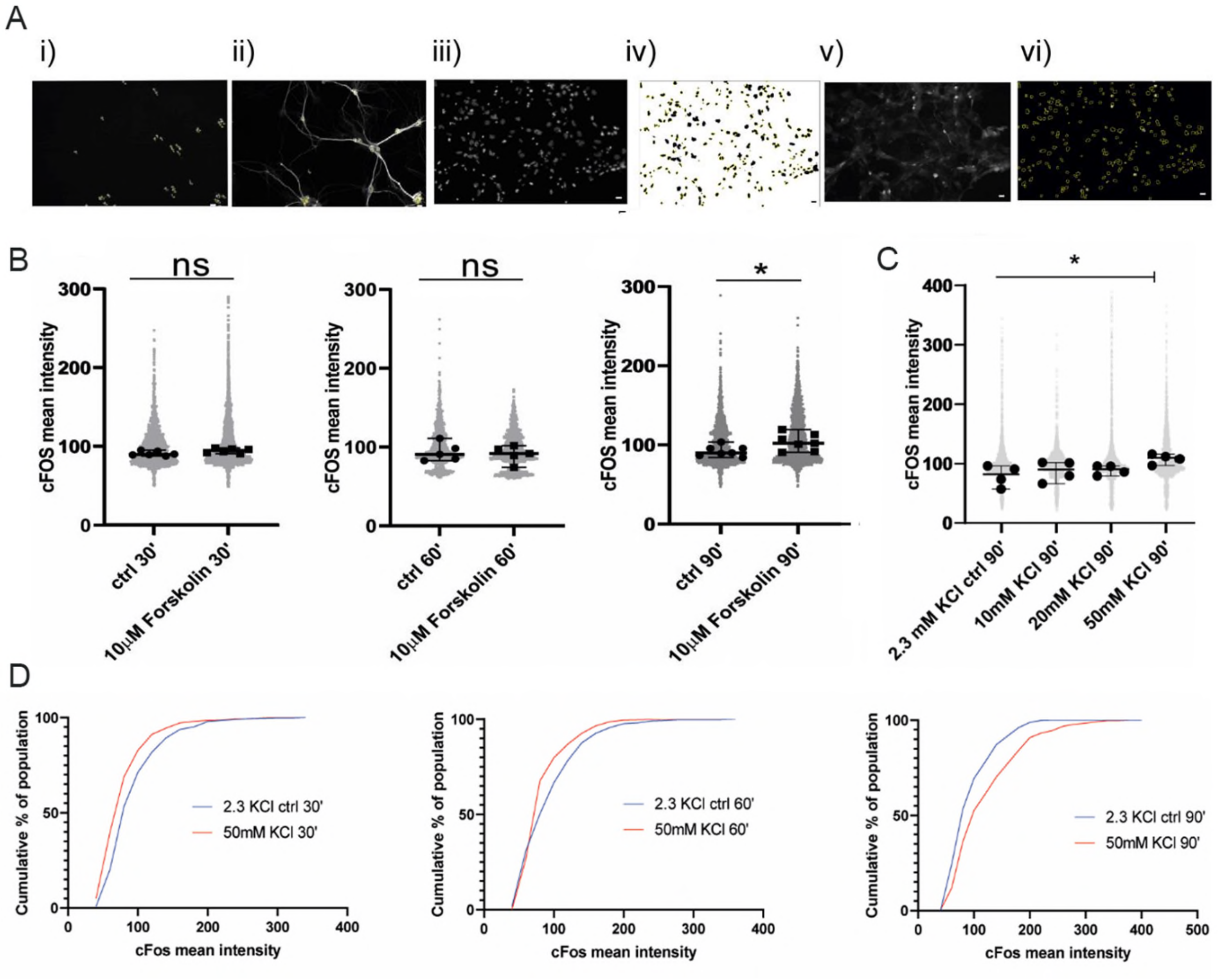

Supplementary Figure 1. c-fos evaluation of cLTP induction and KCl control. A) Representative images illustrating the procedure to create the MACRO for the detection of c-fos positive neurons. Scale bar 10 μm. In i) DAPI+ cells are shown. Only DAPI+ cells with a surface area in the range of 5-10 μm² were taken into account after a threshold application. (ii) DAPI+cells were verified to be neuronal cells because the thresholded DAPI+ cells were super-imposed to a MAP2 staining. iii) Then, a mask of DAPI+ cells was created, and iv) the mask was then applied to c-fos channel image. B) Quantification of the mean intensity for the c-fos stained cells. The c-fos fluorescence intensity was significantly higher after 90 min (ctrl vs cLTP p=0.0379), but not at 30 min (ctrl vs cLTP p=0.0649) or 60 min (n=4 independent cultures, between 900 and 3000 cells analysed for each condition). C). Neurons activated with different concentration of KCl 10 mM, 20 mM and 50 mM. D) Cumulative plots of the fluorescence intensity of neurons for control and KCl 50 mM at 30, 60 and 90 min. The cumulative curve of KCl is shifted to left at 30 and 60 min, showing that the population of neurons has lower fluorescence intensity compared to the control. After KCl 90 min, the cumulative curve of KCl is shifted to right showing that the population of neurons has higher fluorescence intensity compared to the control. ANOVA followed by Holm Sidak’s multiple comparison revealed a significant increment of c-fos intensity after 90 min with 50 mM KCl. n=4 independent experiments.

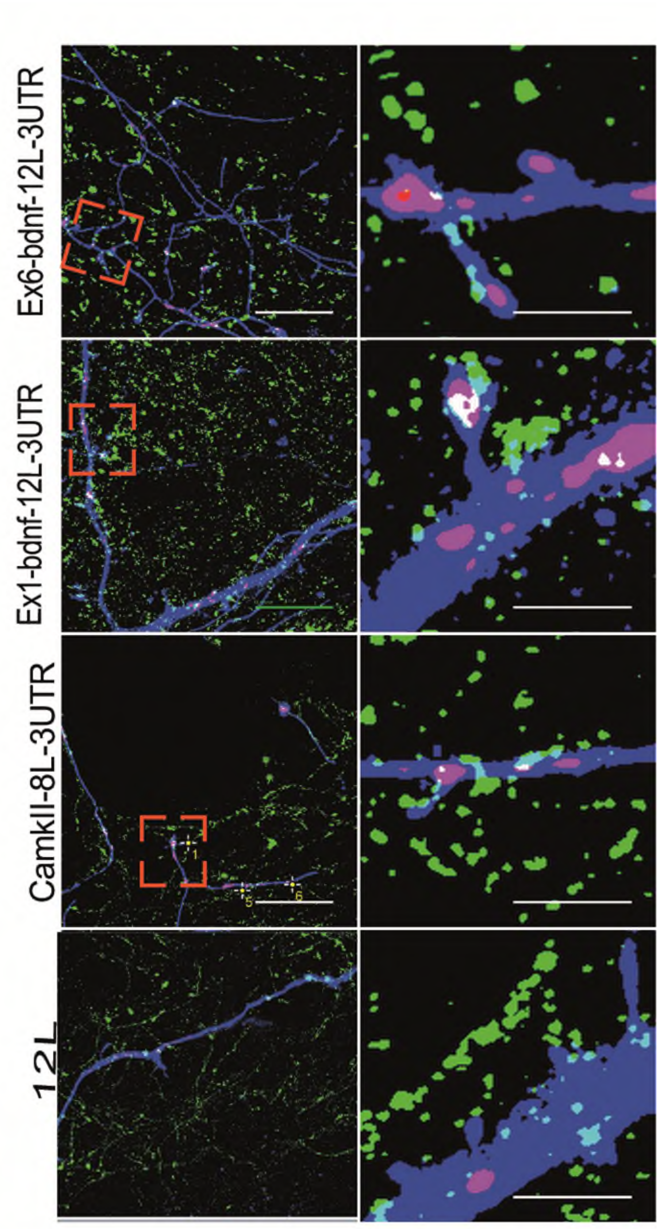

Supplementary Figure 2. Localization of mRNA granules in proximity to dendritic spines. A) Representative thresholded images of all the constructs analysed are show in the low magnification panels on the left. The right panels show high magnification images of the spines and parental dendritic shaft. The mRNA granules for the indicated constructs are in magenta, Synapsin-1 immunofluorescence labelling is shown in green, white spots indicate sites of co-localization of mRNA granules and Synapsin-1. For quantification, granules found in the range of +3 μm and -3 μm were considered. Scale bar 2 μm.

KCL	10mM	20 mM	50 mM
NaCl	131 mM	122 mM	93 mM
$MgS0_4$	0.8 mM	0.8 mM	0.8 mM
$CaCl_2$	1.3mM	1.3mM	1.3mM
Na_2HP0_4	0.35 mM	0.35 mM	0.35 mM
$NaHCO_3$	4.2 mM	4.2 mM	4.2 mM
K_2HPO_4	0.45 mM	0.45 mM	0.45 mM
HEPES	0.5 mM	0.5 mM	0.5 mM
D-glucose	5.5 mM	5.5 mM	5.5 mM

Supplementary Table 1. Composition of solutions containing 10, 20 or 50 mM KCl.

frontiers
